# Supplementary material for: Complete genome of the Medicago anthracnose fungus, Colletotrichum destructivum, reveals a mini-chromosome-like region within a core chromosome
Source: Microb Genom. 2024 Aug 21;10(8):001283. doi: 10.1099/mgen.0.001283 (PMC11338638; doi:10.1099/mgen.0.001283)
Supplement: Uncited Supplementary Material 1. [file mgen-10-01283-s001.pdf]

Supplementary information for

Complete genome of the Medicago anthracnose fungus, *Colletotrichum destructivum*, reveals a mini-chromosome-like region within a core chromosome

Nicolas Lapalu[1§], Adeline Simon[1§], Antoine Lu[1], Peter-Louis Plaumann[2], Joëlle Amselem[3], Sandrine Pigné[1], Annie Auger[1], Christian Koch[2], Jean-Félix Dallery[1#], Richard J. O'Connell[1#]

[1] Université Paris-Saclay, INRAE, UR BIOGER, 91120 Palaiseau, France

[2] Division of Biochemistry, Department of Biology, Friedrich-Alexander-Universität Erlangen-Nürnberg, 91058 Erlangen, Germany

[3] Université Paris-Saclay, INRAE, URGI, 78000 Versailles, France

[§] These authors contributed equally.

[#] **Corresponding authors:** Richard J. O'Connell, richard.oconnell@inrae.fr; Jean-Félix Dallery jean-felix.dallery@inrae.fr.

Table S1: List of *Medicago truncatula* accessions used in this study and their infection phenotypes with *Colletotrichum destructivum* LARS 709.

Accession ID	Hapmap ID	Country of origin	Infection phenotype
DZA016-F	HM048	Algeria	Resistant
DZA210-5	HM083	Algeria	Susceptible
DZA327-7	HM011	Algeria	Susceptible
ESP074-A	HM056	Spain	Susceptible
ESP155-D	HM057	Spain	Susceptible
ESP163-E	HM058	Spain	Resistant
R108-C3*	HM029	Israel	Resistant
SA03648	HM068	Portugal	Susceptible
SA12451	HM075	Italy	Resistant

* R108-C3 (R108) was derived by *in vitro* selection from the natural accession 108-1 (Hoffmann et al. 1997) and is now considered to be *M. truncatula* ssp. *tricycla*.

Table S2: *Colletotrichum spp.* mitochondrial genomes used as reference for assembly of the *C. destructivum* mitochondrial genome with Organelle_PBA

Species/Strain	Size (bp)	Accession number
C. acutatum KC05	30892	NC_027280.1/KR349346.1
C. fioriniae	30020	NC_030052.1/KU375885.1
C. graminicola M1.001	39649	MT: NW_007361658.1/CM001021.1
C. lindemuthianum	36957	NC_023540.1/KF953885.1
C. lupini CBS 119142	36554	NC_029213.1/KT918406.1
C. salicis	33950	NC_035496.1/KY774449.1
C. tamarilloi	30824	NC_029706.1/KU196965.1
C. lindemuthianum isolate 89 A2 2-3	37446	MF595869.1
C. lindemuthianum isolate 83.501	37440	MF595868.1
C. aenigma XY15	57252	KX885105.1
C. gloeosporioides LQ33	55169	KX885104.1
C. siamense YT02	53317	KX885103.1
C. siamense SQ01	54645	KX885102.1
C. siamense LQ22	58666	KX885101.1
C. siamense ZH01	52671	KX885100.1
C. siamense ZH03	54658	KX885099.1
C. siamense ZH02	54679	KX885098.1
C. fructicola	56051	KX034082.1

Table S3: Classification of transposable element (TE) consensus sequences identified in the *Colletotrichum destructivum* genome. Sequences were classified according to [28]. The number and mean length of the different consensus sequences (i.e. non-redundant sequences) per TE superfamily are indicated. The total number of copies in the genome, as well as the number of complete copies, and the corresponding genome coverages were computed. LTR: long terminal repeat, LINE: long interspersed element, TIR: terminal inverted repeat, MITE: miniature inverted-repeat transposable element.

Order	Superfamily	Wicker Code	Number of consensus	Length consensus in bases	Number of copies	Coverage in Kb (%)	Number of complete copies	Coverage by complete copies in Kb (%)
Class I (retrotransposons)								
LTR	Copia	RLC	4	5970 (+/- 1864)	153	301 (0.58%)	39	245 (0.47%)
	Gypsy	RLG	7	10762 (+/- 5480)	719	1489 (2.88%)	106	699 (1.35%)
LINE	I	RII	6	5968 (+/- 881)	289	508 (0.98%)	61	350 (0.68%)
	Other	RIX	1	2598	12	7 (0.01%)	1	3 (0.01%)
Class II (DNA transposons)								
TIR	Tc1-Mariner	DTT	18	1877 (+/-15)	401	355 (0.69%)	136	255 (0.49%)
	hAT	DTA	3	2545 (+/- 921)	67	109 (0.21%)	28	83 (0.16%)
	PiggyBac	DTB	2	2246 (+/- 50)	60	52 (0.10%)	18	41 (0.08%)
	PIF-Harbinger	DTH	1	3061	30	22 (0.04%)	5	15 (0.03%)
	Other	DTX	1	721	7	3 (0.01%)	4	3 (0.01%)
Helitron		DHX	1	11678	56	280 (0.54%)	22	257 (0.50%)
MITE		DXX-MITE	2	614 (+/- 302)	26	7 (0.01%)	5	4 (0.01%)
Uncaracterized TEs			3	858 (+/- 342)	95	44 (0.08%)	32	29 (0.06%)
TOTAL			***49***		***1915***	***3177 (6.14%)***	***457***	***1983 (3.83%)***

Table S4: RNA-Seq transcriptome assembly parameters and metrics: StringTie parameters and assembly results for the three RNA-Seq conditions, namely *Colletotrichum destructivum* isolate LARS 709 mycelium grown in Potato Dextrose Broth (PDB) medium, infected *Medicago sativa* cotyledons at 48 hours post-inoculation (hpi) and infected M. sativa cotyledons at 72 hpi.

	**PDB**	**48 hpi**	**72 hpi**
Uniquely mapped reads	93%	2.5%	6.5%
Mean coverage depth (no. of reads)	82	16	33
StringTie no. of reads junction (-j)	10	3	5
TPM threshold	1.88	9.38	4.90
No. of transcripts	16122	13901	15081
No. of genes	15209	13496	14338
Mean transcript length	1469	1169	1482
Standard deviation	1255	952	1148
Median	1134	883	1202
Minimum	150	150	150
Maximum	23615	13099	13082
Mean read coverage	129	21	47
Standard deviation	763	83	258
Median	16	6	12
Minimum	4	3	4
Maximum	38440	3168	24873
Mean TPM	54	63	58
Standard deviation	319	243	317
Median	6	18	15
Minimum	1.88	9.38	4.90
Maximum	16091	9184	30486

RNA-seq libraries were prepared from total RNA isolated from the following samples:

PDB = mycelium grown in potato dextrose broth

48 hpi = infected *Medicago sativa* cotyledons at 48 h post inoculation

72 hpi = infected *Medicago sativa* cotyledons at 72 h post inoculation

TPM = transcripts per million

Table S5: Genome annotation statistics for *Colletotrichum destructivum* isolate LARS 709: Results of structural annotation (gene and transposable element prediction) and functional annotation (Carbohydrate-Active enZymes, candidate secreted effector proteins and secondary metabolism key enzymes).

**Annotation statistics**	
Coverage by Transposable Elements	6.2%
Number of predicted genes	15 631
Number of genes with RNA-Seq support	11 853
Number of genes with protein support	15 172
Average gene length (bp)	1976
Average exon length (bp)	649
Average number of exons per gene	2.79
Average intron length (bp)	88
Average number of introns per gene	1.79
Number of mono-exon genes	4055
Average CDS length (bp)	1378
Annotation completeness (BUSCO)*	
Complete proteins	1309 (99.54%)
Fragmented proteins	5 (0.38%)
Missing proteins	1 (0.08%)
Number of predicted CAZymes	619
Number of predicted effector proteins	484
Number of predicted secondary metabolism key enzymes (SMKEs)	110

* BUSCO = Benchmarking Universal Single-Copy Orthologs

Table S7: Summary of the results of predicting syntenic blocks in *C. destructivum* and *C. higginsianum* using SynChro (delta parameter = 1). The number of syntenic blocks generated from Reciprocal Best-Hits and numbers of associated genes are shown.

	Fungal Genome	
	C. destructivum	***C. higginsianum***
No. of Reciprocal Best-Hits	12135	
Similarity (%)	93.9	
No. of syntenic blocks (SB)	400	
No. of genes in syntenic blocks	14311	13921
Proportion of genome in SB (%)	88.0	91.6

Table S8: List of non-syntenic blocks identified by Synchro in *Colletotrichum destructivum* isolate LARS 709 compared to *Colletotrichum higginsianum* IMI 349063A, where non-syntenic blocks were defined as regions containing at least 5 consecutive genes specific to *C. destructivum*. For each block, the chromosomal location in *C. destructivum*, length and gene content are indicated

Chromosome	Start	End	Length (bp)	No. of genes	Block ID
chr1	4178820	4203721	24901	5	block_0
chr1	4210554	4340246	129692	32	block_1
chr1	4348070	4378433	30363	12	block_2
chr1	4397823	4426916	29093	9	block_3
chr1	4568340	4599600	31260	13	block_4
chr1	6066423	7282629	1216206	305	block_5
chr1	7307926	7321922	13996	6	block_6
chr2	12042	52349	40307	11	block_7
chr2	4605099	4613936	8837	5	block_8
chr2	5997899	6047905	50006	15	block_9
chr2	6062466	6093962	31496	8	block_10
chr3	46529	55509	8980	6	block_11
chr3	723104	737102	13998	6	block_12
chr3	5641079	5662083	21004	12	block_13
chr3	5871897	5895410	23513	7	block_14
chr4	13059	104775	91716	15	block_15
chr4	352058	363077	11019	7	block_16
chr4	752022	769918	17896	8	block_17
chr4	3379899	3389434	9535	5	block_18
chr5	21675	79289	57614	9	block_19
chr5	89463	100537	11074	5	block_20
chr5	1346906	1442665	95759	11	block_21
chr5	4889101	4902562	13461	5	block_22
chr5	5176186	5186138	9952	5	block_23
chr5	5238579	5277212	38633	17	block_24
chr6	13368	75899	62531	18	block_25
chr6	200524	370735	170211	46	block_26
chr6	4320111	4364960	44849	5	block_27
chr7	4117709	4131681	13972	6	block_28
chr8	7311	37105	29794	11	block_29
chr8	3909912	3964560	54648	6	block_30
chr8	4012267	4028388	16121	7	block_31
chr8	4074802	4104606	29804	11	block_32
chr10	11605	31856	20251	6	block_33
chr10	92306	124025	31719	15	block_34
chr10	373355	477333	103978	34	block_35
chr10	1628812	1639928	11116	5	block_36
chr10	1876452	1892426	15974	7	block_37
chr10	2898367	2924158	25791	14	block_38
chr11	12418	20573	8155	6	block_39
chr11	139951	178181	38230	9	block_40
chr11	271755	357039	85284	9	block_41
chr11	378813	604554	225741	38	block_42
chr11	663865	691604	27739	7	block_43
chr11	697412	1175606	478194	106	block_44
chr11	1223231	1263891	40660	7	block_45
chr12	15082	800244	785162	171	block_46

Table S9: Gene ontology enrichment tables of *C. destructivum*-specific genes in blocks that are non-syntenic with *Ch*63 detected with SynChro. No enrichments in Cellular Component (CC) were detected with the topGO R library.

a) Molecular Function

GO.ID	Term	Annotated	Significant	Expected	p-value
GO:0004672	protein kinase activity	187	28	9.51	1.90E-07
GO:0016773	phosphotransferase activity	230	28	11.7	1.30E-05
GO:0046914	transition metal ion binding	992	78	50.45	2.60E-05
GO:0005506	iron ion binding	297	31	15.11	9.10E-05
GO:0016301	kinase activity	257	28	13.07	9.60E-05
GO:0031177	phosphopantetheine binding	57	11	2.9	0.00011
GO:0072341	modified amino acid binding	57	11	2.9	0.00011
GO:0033218	amide binding	65	11	3.31	0.00037
GO:0016772	transferase activity	331	31	16.84	0.00063
GO:0005488	binding	3713	216	188.85	0.00068
GO:0020037	heme binding	301	28	15.31	0.00129
GO:0046906	tetrapyrrole binding	301	28	15.31	0.00129
GO:0043167	ion binding	2217	138	112.76	0.00155
GO:0003700	DNA-binding transcription factor activity	55	9	2.8	0.00163
GO:0046872	metal ion binding	1166	80	59.3	0.00189
GO:0043169	cation binding	1173	80	59.66	0.00225
GO:0140110	transcription regulator activity	77	10	3.92	0.00529
GO:0004834	tryptophan synthase activity	3	2	0.15	0.00748
GO:0008270	zinc ion binding	635	46	32.3	0.00784
GO:0140096	catalytic activity, acting on a protein	542	40	27.57	0.00968

b) Biological Process

GO.ID	Term	Annotated	Significant	Expected	p-value
GO:0006468	protein phosphorylation	184	28	7.98	3.00E-09
GO:0016310	phosphorylation	262	28	11.37	5.90E-06
GO:0009403	toxin biosynthetic process	63	12	2.73	1.20E-05
GO:0009404	toxin metabolic process	63	12	2.73	1.20E-05
GO:0043385	mycotoxin metabolic process	63	12	2.73	1.20E-05
GO:0043386	mycotoxin biosynthetic process	63	12	2.73	1.20E-05
GO:0044550	secondary metabolite biosynthetic process	65	12	2.82	1.70E-05
GO:0019748	secondary metabolic process	69	12	2.99	3.20E-05
GO:0008152	metabolic process	3257	163	141.3	0.00019
GO:0055114	oxidation-reduction process	1053	67	45.68	0.00028
GO:0006464	cellular protein modification process	346	28	15.01	0.00083
GO:0036211	protein modification process	346	28	15.01	0.00083
GO:0043412	macromolecule modification	385	28	16.7	0.0041

Table S10: Functional enrichment test (Fisher's exact test). Complete lists of *C. destructivum* Secondary metabolism key genes (SMKGs), and genes encoding effectors and carbohydrate-active enzymes (CAZYmes) were used to detect enrichments among these functional categories in the 1083 *C. destructivum*-specific genes in non-syntenic blocks detected using SynChro.

	In genome	In non-syntenic blocks	Odds ratio	*p*-value
Total No. of genes	15631	1083		
No. of SMKGs	123	14	1.6	8.16E-02
No. of effector genes	484	49	1.5	1.62E-02
No. of CAZyme genes	619	14	0.3	1.62E-06

Table S11: Comparison of the GC content, gene content and transposable element (TE) content for each *Colletotrichum destructivum* chromosome, including overall results for the entire chromosome 1 and for the regions 1A and 1B of chromosome 1.

Chr	Size (bp)	No. genes	GC (%)	TE content %)	Telomeres
1	7333624	2172	54.60	8.7	2
1A	*6127957*	*1872*	*55.10*	*4.0*	*2*
1B	*1205667*	*300*	*52.30*	*32.8*	*NA*
2	6118321	1884	54.69	3.8	2
3	5939657	1874	55.06	4.1	2
4	5487742	1666	55.09	3.9	2
5	5290227	1573	54.75	4.3	2
6	4379568	1360	54.01	5.5	2
7	4147912	1312	54.54	5.0	2
8	4124671	1252	53.98	6.2	2
9	3904609	1167	55.26	3.0	2
10	2936318	922	54.23	4.6	2
11	1275594	278	51.32	32.3	2
12	812569	171	50.22	35.1	2

TE = transposable element

Table S13: List of chromosomal location pairs of the eight largest segmentally duplicated regions present in the chr1B region of chromosome 1 of *Colletotrichum destructivum*, detected by SDDetector from sequence alignments with a minimum of 90% sequence similarity.

Code	chr	Start	End	Duplication	Length (bp)	Pairing
SD1B-1	chr1B	6275896	6310837	chr1:6275896-6309509 // chr1:7126431-7156805	28087	SD1B-1 // SD1B-7
				chr1:6275896-6310837 // chr1:6440881-6474186	30727	SD1B-1 // SD1B-3
SD1B-2	chr1B	6423690	6434928	chr1:6423690-6434928 // chr1:7113913-7125518	11316	SD1B-2 // SD1B-6
SD1B-3	chr1B	6440881	6476743	chr1:6275896-6310837 // chr1:6440881-6474186	30727	SD1B-3 // SD1B-1
				chr1:6459979-6476743 // chr1:7145173-7159363	14169	SD1B-3 // SD1B-7
				chr1:6442213-6459984 // chr1:7126431-7143900	16220	SD1B-3 // SD1B-7
				chr1:6442842-6464026 // chr1:6844058-6866689	19709	SD1B-3 // SD1B-5
SD1B-4	chr1B	6481996	6501507	chr1:6481996-6501507 // chr1:7201724-7220918	16885	SD1B-4 // SD1B-8
SD1B-5	chr1B	6844058	6866689	chr1:6846262-6862419 // chr1:7129446-7147001	15244	SD1B-5 // SD1B-7
				chr1:6442842-6464026 // chr1:6844058-6866689	19709	SD1B-5 // SD1B-3
SD1B-6	chr1B	7113913	7125518	chr1:6423690-6434928 // chr1:7113913-7125518	11316	SD1B-6 // SD1B-2
SD1B-7	chr1B	7126431	7159363	chr1:6275896-6309509 // chr1:7126431-7156805	28087	SD1B-7 // SD1B-1
				chr1:6846262-6862419 // chr1:7129446-7147001	15244	SD1B-7 // SD1B-5
				chr1:6442213-6459984 // chr1:7126431-7143900	16220	SD1B-7 // SD1B-3
				chr1:6459979-6476743 // chr1:7145173-7159363	14169	SD1B-7 // SD1B-3
SD1B-8	chr1B	7201724	7220918	chr1:6481996-6501507 // chr1:7201724-7220918	16885	SD1B-8 // SD1B-4

Table S14: Number of proteins conserved between *Colletotrichum destructivum* (Cd709) and twenty-three other *Colletotrichum* species identified by Blastp protein alignments with thresholds of 30% amino acid identity and 50% coverage. Data are shown for the total *C. destructivum* proteome (15631 proteins) and for the chromosome 1 region Chr1B (300 proteins).

	***Cd*709 total proteome (15631)**		***Cd*709 Chr1B Proteome (300)**	
Species	Retrieved	%*	Retrieved	%*
C. chlorophyte	11098	71.0	85	28.3
C. fioriniae	12339	78.9	108	36.0
C. fructicola	11852	75.8	165	55.0
C. gloeosporioides	11666	74.6	84	28.0
C. graminicola	11798	75.5	93	31.0
C. higginsianum	14372	91.9	134	44.7
C. incanum	12700	81.2	173	57.7
C. musicola	12370	79.1	136	45.3
C. nymphaeae	12443	79.6	177	59.0
C. orbiculare	12091	77.4	92	30.7
C. orchidophilum	12381	79.2	203	67.7
C. plurivorum	12352	79.0	121	40.3
C. salicis	12171	77.9	164	54.7
C. shisoi	12644	80.9	92	30.7
C. sidae	11447	73.2	89	29.7
C. simmondsii	12361	79.1	103	34.3
C. sojae	12362	79.1	120	40.0
C. spinosum	11520	73.7	88	29.3
C. sublineola	12107	77.5	202	67.3
C. tanaceti	12183	77.9	94	31.3
C. tofieldiae	12210	78.1	142	47.3
C. trifolii	11481	73.5	84	28.0
C. truncatum	12561	80.4	217	72.3
All species	15081	96.4	278	92.6

* % of completeness of the whole set of protein compared to *Cd*709 (Blastp cut-offs 30% identity, 50% coverage).

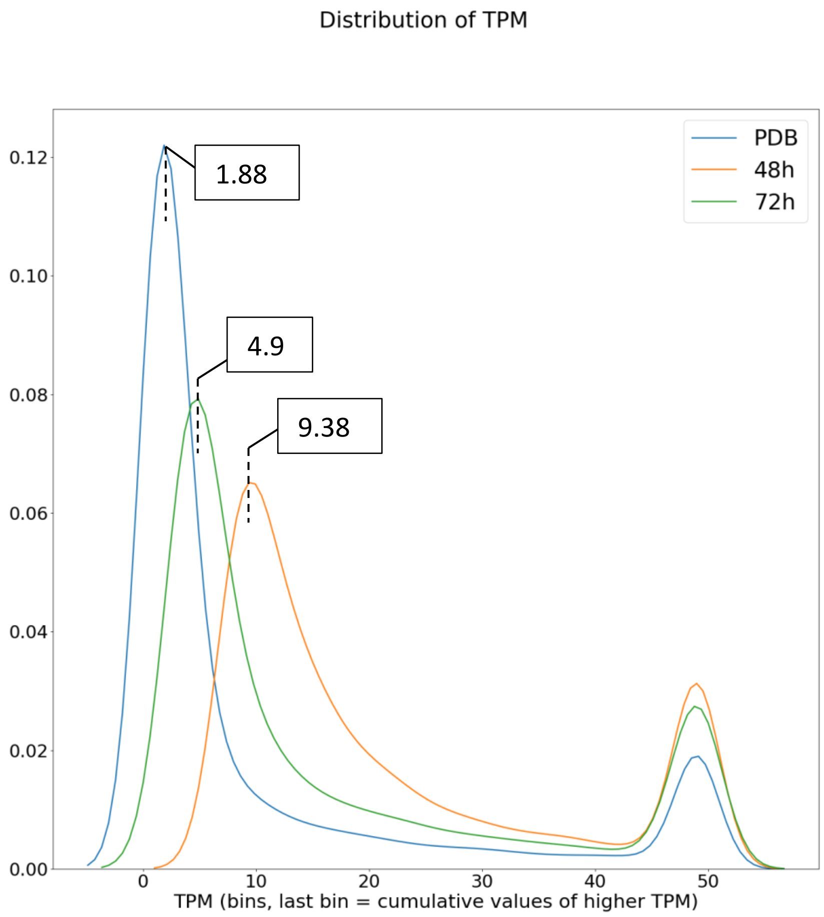

Figure S1: Distribution of Transcript Per Million (TPM) for transcripts assembled from the three RNA-Seq conditions, namely *Colletotrichum destructivum* mycelia grown in potato dextrose broth (PDB) and infected *Medicago sativa* cotyledons at 48 hours post-inoculation (hpi) and 72 hpi. Thresholds used to filter out assembled transcripts lacking sufficient coverage with RNA-Seq reads are indicated for each of the conditions.

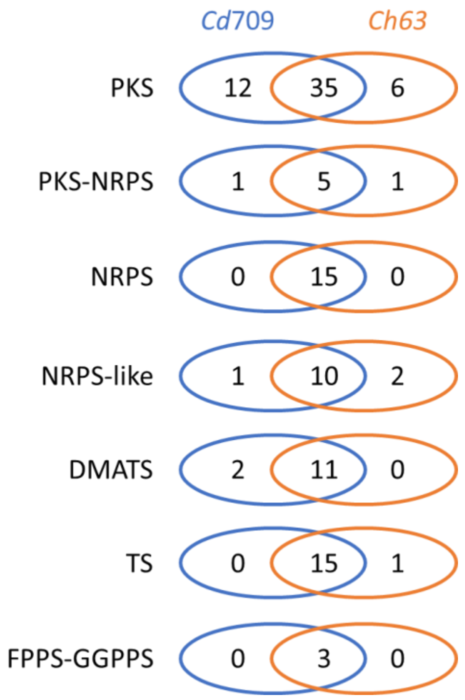

Figure S2: Number of genes encoding secondary metabolism key genes (SMKG) in *Colletotrichum destructivum* (*Cd*709) and *Colletotrichum higginsianum* (*Ch*63) genomes. Genes were those predicted as biosynthetic SMKG by Dallery et al. (2017) for *Ch*63 or by antiSMASH for *Cd*709. *Cd*709 genes not predicted as SMKG by antiSMASH, but orthologous to a *Ch*63 SMKG were included. PKS: Polyketide Synthase, NRPS: Nonribosomal Peptide Synthetase, DMATS: Dimethylallyltryptophan synthase, TS: Terpene Synthase, FPPS: Farnesyl pyrophosphate sythase, GGPPS: Geranylgeranyl pyrophosphate synthase

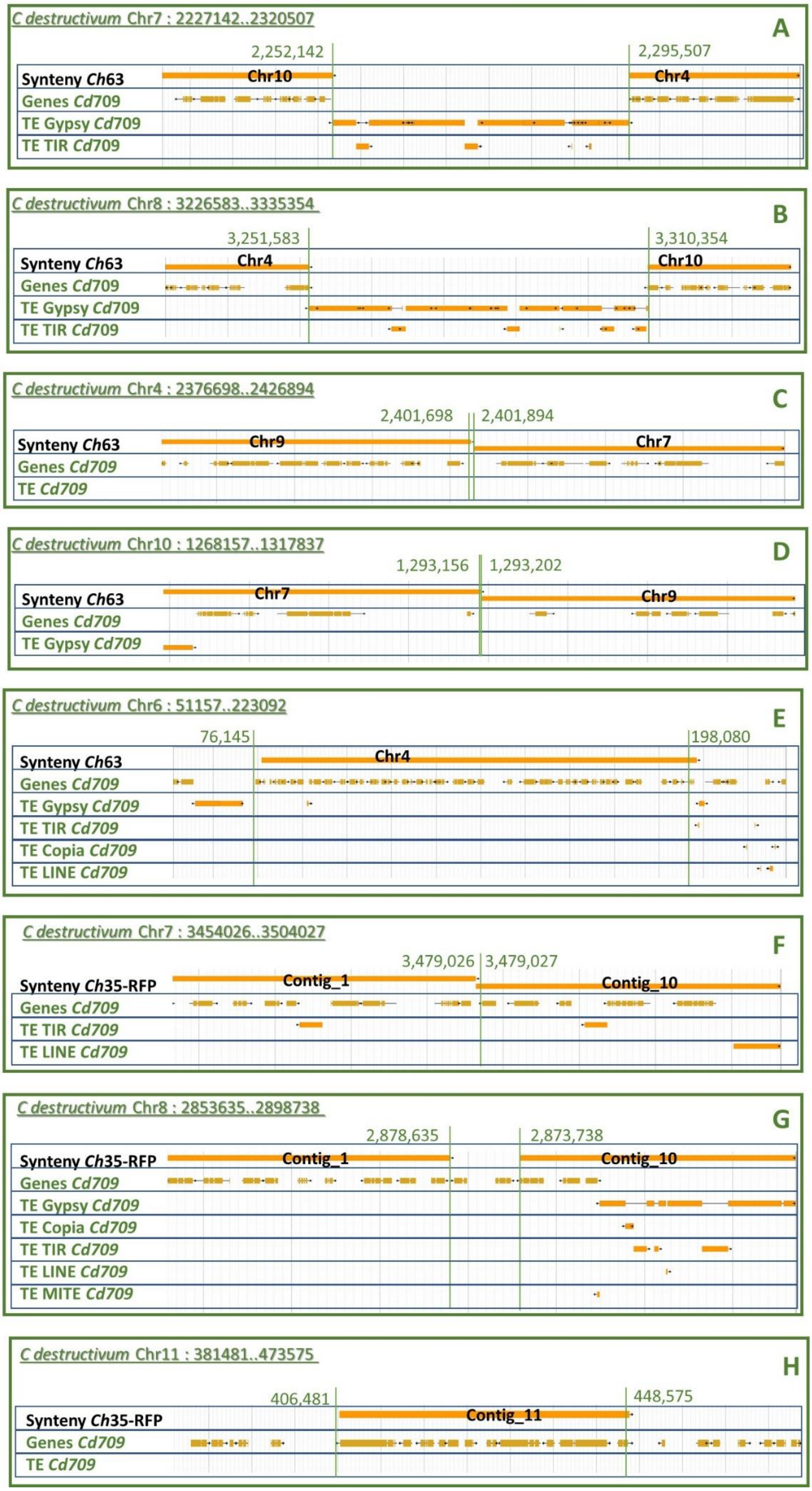
C destructivum Chr7 : 2227142..2320507
A
2,252,142
2,295,507
Synteny Ch63
Chr10
Chr4
Genes Cd709
TE Gypsy Cd709
TE TIR Cd709
C destructivum Chr8 : 3226583..3335354
B
3,251,583
3,310,354
Synteny Ch63
Chr4
Chr10
Genes Cd709
TE Gypsy Cd709
TE TIR Cd709
C destructivum Chr4 : 2376698..2426894
C
2,401,698
2,401,894
Synteny Ch63
Chr9
Chr7
Genes Cd709
TE Cd709
C destructivum Chr10 : 1268157..1317837
D
1,293,156
1,293,202
Synteny Ch63
Chr7
Chr9
Genes Cd709
TE Gypsy Cd709
C destructivum Chr6 : 51157..223092
E
76,145
198,080
Synteny Ch63
Chr4
Genes Cd709
TE Gypsy Cd709
TE TIR Cd709
TE Copia Cd709
TE LINE Cd709
C destructivum Chr7 : 3454026..3504027
F
3,479,026
3,479,027
Synteny Ch35-RFP
Contig_1
Contig_10
Genes Cd709
TE TIR Cd709
TE LINE Cd709
C destructivum Chr8 : 2853635..2898738
G
2,878,635
2,873,738
Synteny Ch35-RFP
Contig_1
Contig_10
Genes Cd709
TE Gypsy Cd709
TE Copia Cd709
TE TIR Cd709
TE LINE Cd709
TE MITE Cd709
C destructivum Chr11 : 381481..473575
H
406,481
448,575
Synteny Ch35-RFP
Contig_11
Genes Cd709
TE Cd709

Figure S3: Genomic context of the large-scale rearrangements found between the chromosomes of *Colletotrichum destructivum* (*Cd*709) and *Colletotrichum higginsianum* IMI 349063 (*Ch*63) or MAFF 305635 (*Ch*35-RFP). A region of 25 kb surrounding each rearrangement zone is depicted. The synteny of each *C. destructivum* chromosome region with *C. higginsianum* IMI 349063 or MAFF 305635 is displayed, as well as predicted genes and transposable elements (TE).

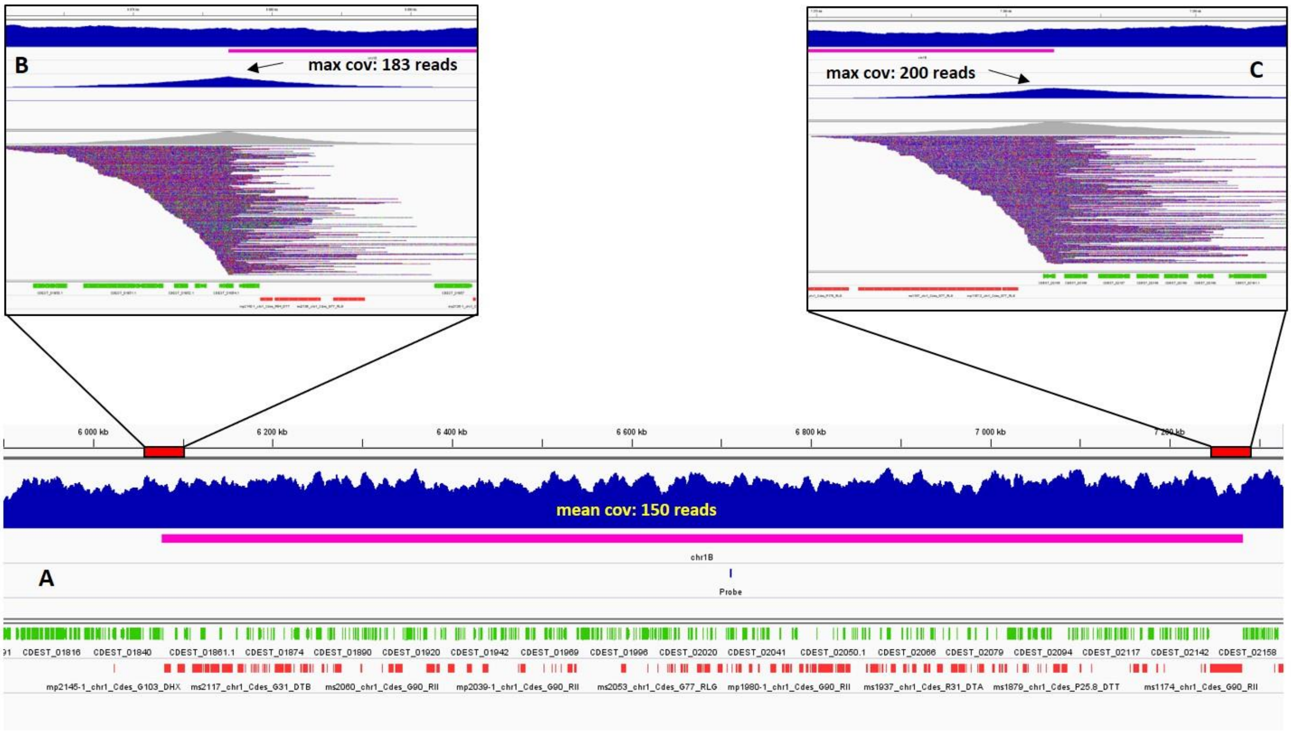

Figure S4: Integrative Genome Viewer screenshot of *Colletotrichum destructivum* chromosome 1 region chr1B, showing uniform coverage by PacBio long read sequences along the entire region and across the two junctions with the rest of chromosome 1 (Chr1A). The chr1B region is shown in pink. Genes and transposable elements are shown in green and red, respectively. The read coverage is shown in blue. Long reads spanning the last base of the 5’ junction (B) and 3’ junction (C) of the chr1B region were extracted from the mapping to verify the support for each junction.

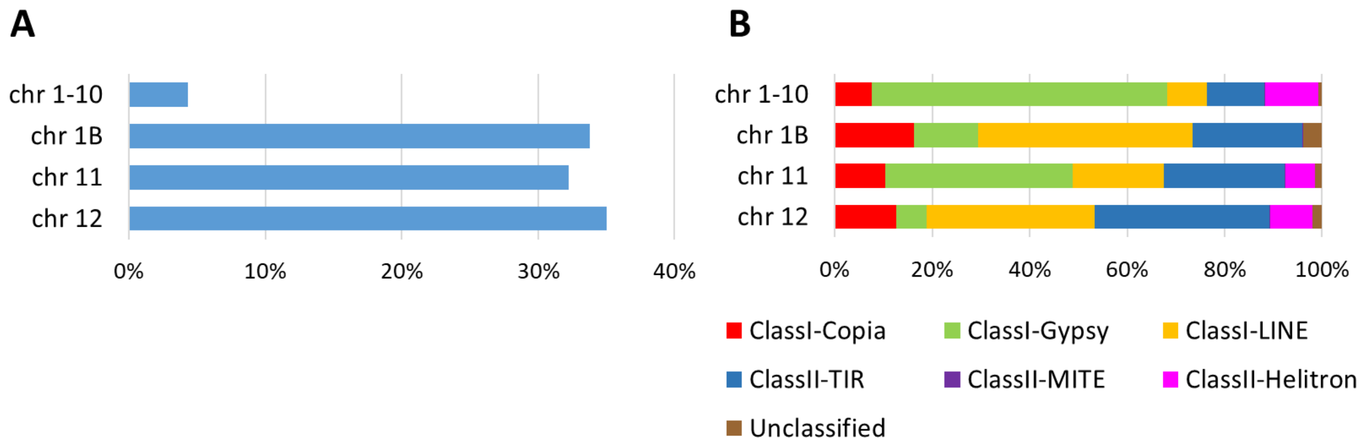

Figure S5: Transposable element (TE) content of the core chromosomes (1-10, excluding 1B), mini-chromosomes 11 and 12 and region 1B of *Colletotrichum destructivum* (*Cd*709). (A) Histogram showing TE percent coverage by length for each chromosome compartment. **(B)** Histogram showing percent coverage by length of the identified TE orders or superfamilies for each chromosome compartment.

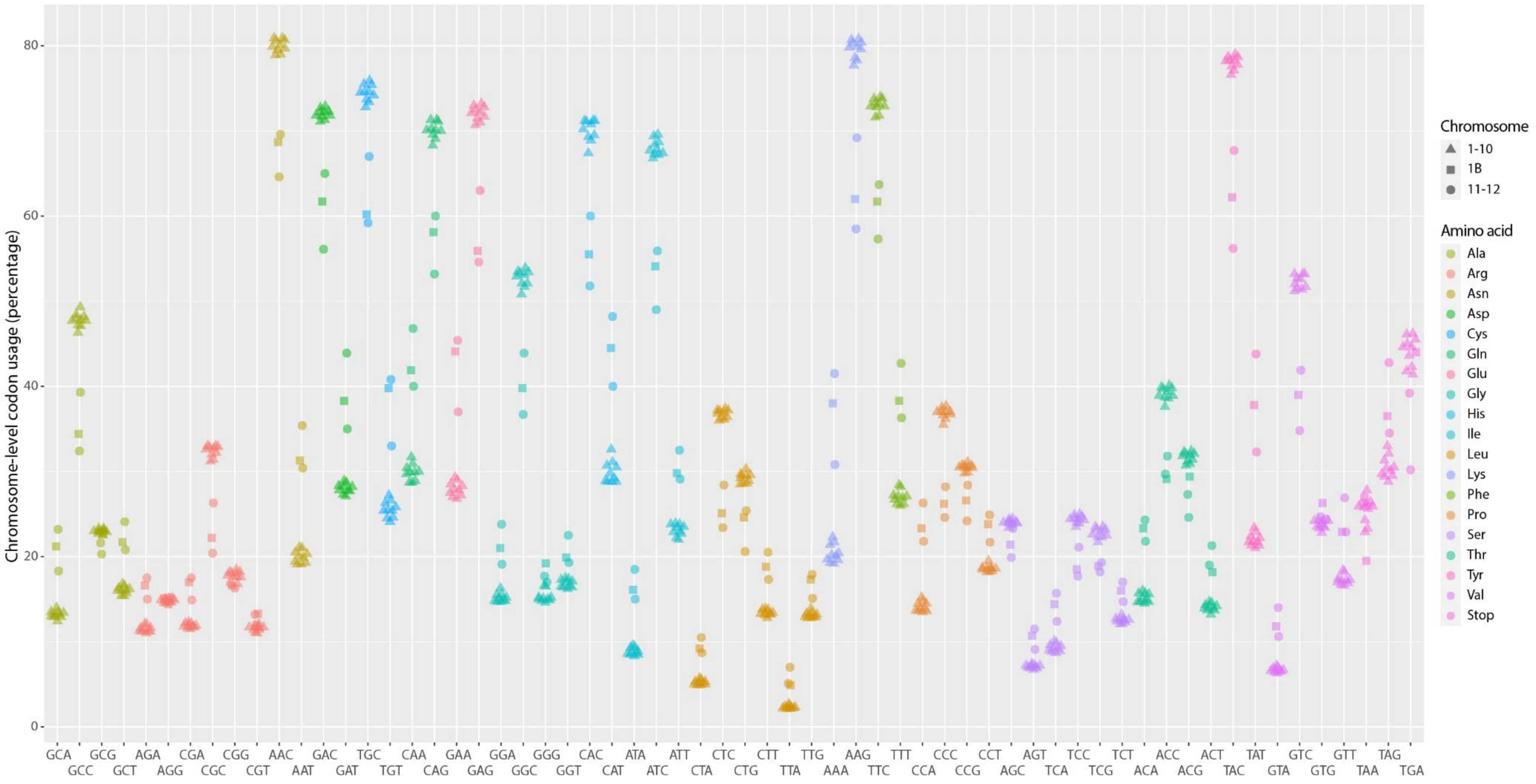

Figure S6: Codon usage bias in core chromosomes compared to mini chromosomes of *Colletotrichum destructivum* LARS709. The 18 amino acids and their 59 corresponding codons are represented.

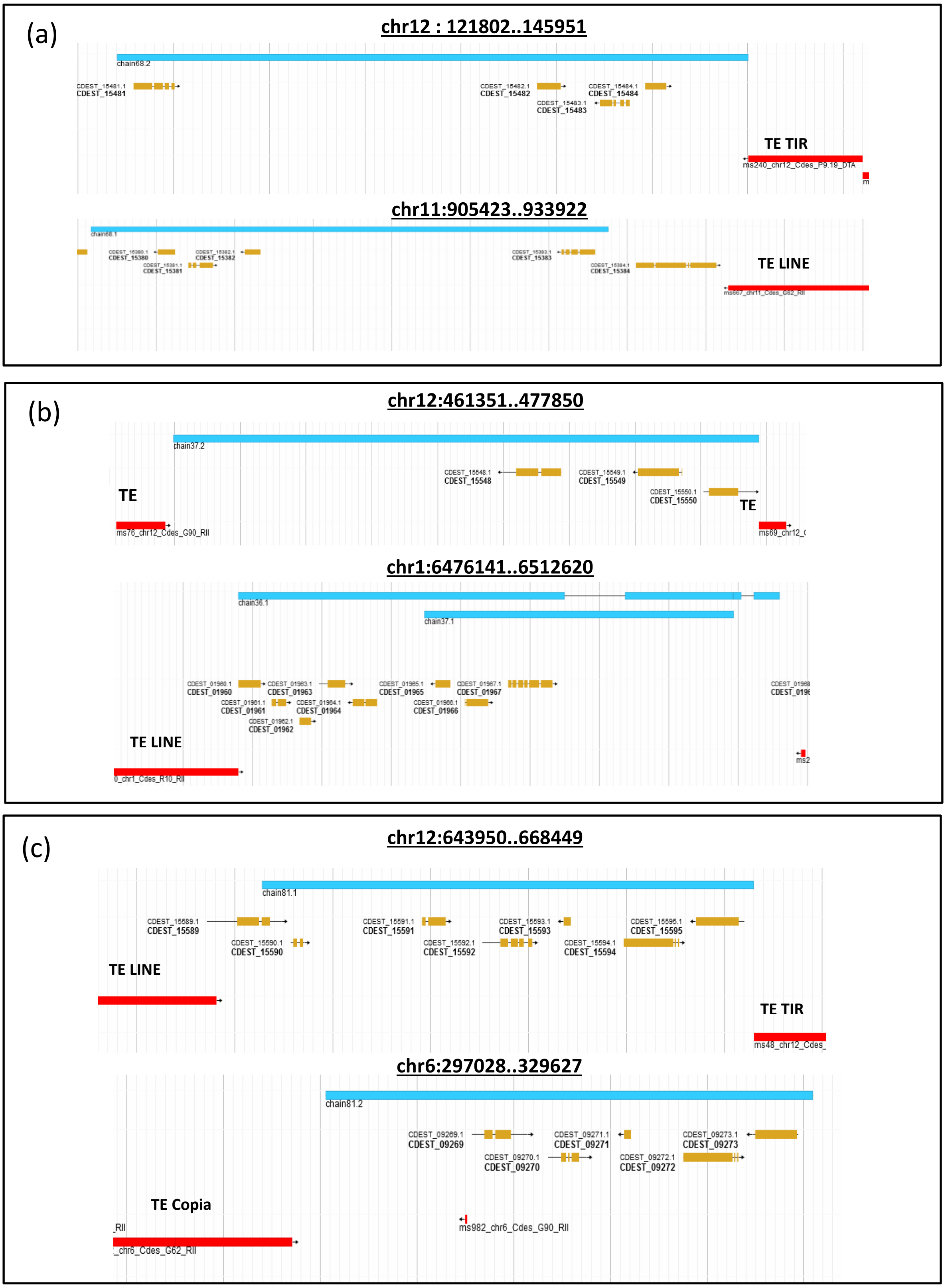

Figure S7: The inter-chromosomal segmental duplications regions detected in *Colletotrichum destructivum* (*Cd*709) and their surrounding genomic regions rich in Transposable Elements (TE).

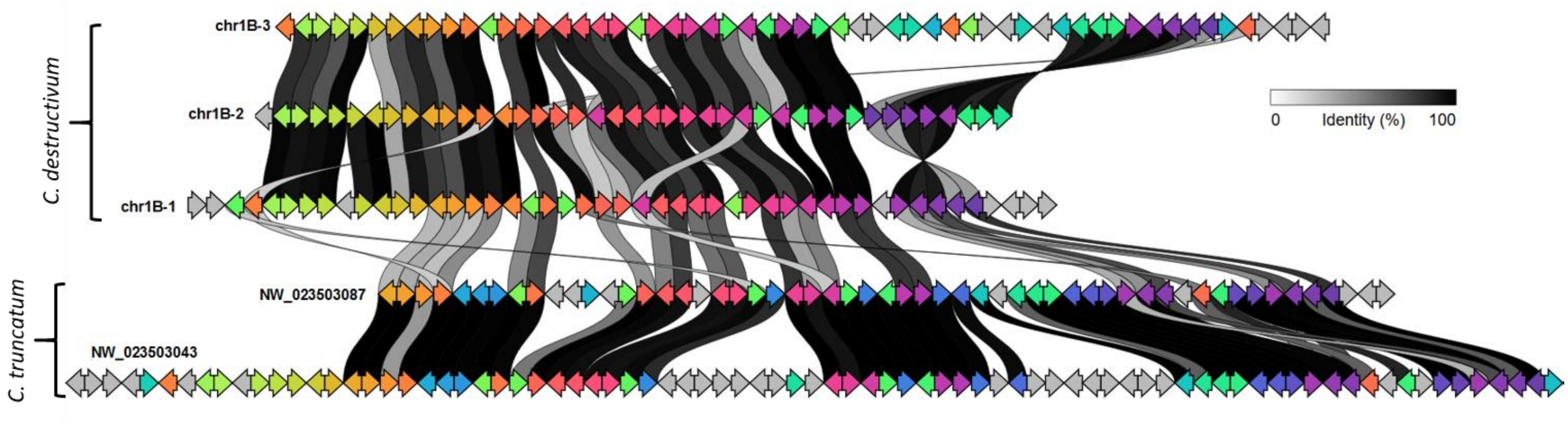

Figure S8: Synteny of segmentally duplicated regions in *Colletotrichum destructivum* chr1B and *Colletotrichum truncatum* CMES1059 contigs.

The duplicated regions chr1B-1 (chr1: 6242820..6372217), chr1B-2 (chr1: 6381352..6493327) and chr1B-3 (chr1: 7087658..7243846) were aligned at the protein level against the *C. truncatum* contigs NW_02350387 (218796..352904) and NW_023503043 (1580..217244). The chr1B-1 region contains the duplication SD1B-1 and adjacent genes (CDEST_1880 to CDEST_1926), the chr1B-2 region contains SD1B-2, SD1B-3 and SD1B-4 (CDEST_1927 to CDEST_1967) and the chr1B-3 contains SD1B-6, SD1B-7 and SD1B-8 (CDEST_2098 to CDEST_2154). The synteny map generated by Clinker shows a strong intra-species similarity and more divergence between the two species.
